# Umbelliprenin is cytotoxic against QU-DB large cell lung cancer cell line but anti-proliferative against A549 adenocarcinoma cells

**DOI:** 10.1186/2008-2231-20-69

**Published:** 2012-10-30

**Authors:** Narges Khaghanzadeh, Zahra Mojtahedi, Mohammad Ramezani, Nasrollah Erfani, Abbas Ghaderi

**Affiliations:** 1 Shiraz Institute for Cancer Research, Shiraz University of Medical Sciences, School of Medicine, Shiraz, Iran; 2 Department of Immunology, Shiraz University of Medical Sciences, School of Medicine, Shiraz, Iran; 3 Pharmaceutical Research Center, School of Pharmacy, Mashhad University of Medical Sciences, Mashhad, Iran

**Keywords:** Annexin, IC_50_, Lung cancer, MTT assay, Propidium Iodide, Umbelliprenin

## Abstract

**Background:**

Umbelliprenin is a natural compound, belonging to the class of sesquiterpene coumarins. Recently, umbelliprenin has attracted the researchers' attention for its antitumor activities against skin tumors. Its effect on lung cancer is largely unknown. The aim of our study was to investigate the effects of this natural compound, which is expected to have low adverse effects, on lung cancer.

**Methods:**

The QU-DB large cell and A549 adenocarcinoma lung cancer cell lines were treated with umbelliprenin. IC_50_ values were estimated using methyl thiazolely diphenyl-tetrazolium bromide (MTT) assay, in which a decrease in MTT reduction can occur as a result of cell death or cell proliferation inhibition. To quantify the rate of cell death at IC_50_ values, flow cytometry using Annexin V-FITC (for apoptotic cells), and propidium iodide (for necrotic cells) dyes were employed.

**Results:**

Data from three independent MTT experiments in triplicate revealed that IC_50_ values for QU-DB and A549 were 47 ± 5.3 μM and 52 ± 1.97 μM, respectively. Annexin V/PI staining demonstrated that umbelliprenin treatment at IC_50_ induced 50% cell death in QU-DB cells, but produced no significant death in A549 cells until increasing the umbelliprenin concentration to IC_80_. The pattern of cell death was predominantly apoptosis in both cell lines. When peripheral blood mononuclear cells were treated with 50 μM and less concentrations of umbelliprenin, no suppressive effect was observed.

**Conclusions:**

We found cytotoxic/anti-proliferative effects of umbelliprenin against two different types of lung cancer cell lines.

## Background

Lung cancer is one of the most common causes of cancer related deaths in most countries [1]. In Iran, lung cancer is the third most frequent cancer in both men and women [2]. The current 5 year survival of lung cancer is about 10–15% mostly being attributed to late diagnosis and inefficient therapy [3,4]. The limited success and significant side effects of lung cancer treatment with classic chemotherapeutic agents has led researchers to find more efficient drugs with fewer side effects [5,6].

Umbelliprenin is synthesized by various *Ferula* plant species such as *Citrus Limon*[7]. It is a prenylated compound that belongs to the class of sesquiterpene coumarins, and possesses a similar structure to auraptene, a compound whose antitumor activities have largely been investigated [8]. Recently, umbelliprenin has attracted the researchers' attention for its antitumor activities. It has been shown that the cell susceptibility to umbelliprenin decreased in the order M4Beu (metastatic pigmented malignant melanoma) > A549 (non-small cell lung carcinoma) ≈ PC3 (androgen resistant prostate carcinoma) > PA1 (ovary teratocarcinoma) > human primary fibroblasts [9]. The anti-tumor effect of umbelliprenin has also been demonstrated in vivo in a mouse skin tumor model in which oral administration of umbelliprenin significantly delayed the formation of papilloma [10]. Umbelliprenin also showed an inhibitory effect on the matrix metalloproteinases activity [11]. Matrix metalloproteinases are critical enzymes in cancer metastatic cascade, such as migration, angiogenesis, and invasiveness [12,13]. Despite inhibitory effects of umbelliprenin on tumor cells, treatment of peripheral blood mononuclear cells (PBMNCs) with this compound protected them against DNA and chromosome damage induced by H_2_O_2_[12-14]^.^

Methyl thiazolely diphenyl-tetrazolium bromide (MTT) assay is frequently employed to determine the in vitro index of cytotoxicity (IC). In metabolically active cells, the reduction of water soluble MTT into an insoluble purple formazan results in accumulation of purple formazan crystals in cells. After solubilization of these crystals, the comparison of the optical density (OD) between treated and untreated cells gives a relative estimation of IC at different doses [15]. For determination of whether decreased MTT reduction is either due to the cell death or decrease in cell proliferation, other complementary tests such as staining with fluorescent dyes annexin V-FITC for apoptotic cells and propidium iodide (PI) for necrotic cells are frequently performed. The sum of apoptotic and necrotic cells represents the total rate of cell death [16].

The effect of umbelliprenin on lung cancer is poorly understood. Here, we employed MTT assay and annexin V/PI staining to investigate the effects of umbelliprenin on lung cancer cells. The A549, a human pulmonary adenocarcinoma cell line, and QU-DB, a human large cell carcinoma line, as well as PBMNCs were treated with umbelliprenin. IC_50_ was defined as the umbelliprenin concentration that resulted in a 50% decrease in the OD of the test compound compared to that of untreated cells. Then, annexin V/PI staining determined whether (i) the decrease in MTT reduction is due to the cell death or suppression of cell proliferation, and (ii) apoptosis or necrosis is the main cause of cell death.

## Materials and methods

### Cell culture

The study was approved by the Ethic Committee of Shiraz University of Medical Sciences. Two human lung cancer cell lines including A549 (adenocarcinoma) and QU-DB (large cell) were obtained from the National Cell Bank of Iran (NCBI), Pasteur Institute of Iran, Tehran, Iran. PBMCs were obtained from healthy volunteers by Ficoll-Hypaque density sedimentation. All the cells were cultured in RPMI-1640 medium supplemented with 10% heat inactivated fetal bovine serum (FBS) (Gibco/BRL, Germany), penicillin 100 U/ml. and streptomycin 100 μg/ml (Biosera, UK). They were maintained at 37°C in a 5% CO_2_ incubator and the media were changed every two day.

### Isolation of human peripheral blood mononuclear cells

Participants were informed that blood samples will be used for a research project as healthy individuals, and their written consent was obtained. Fifteen ml heparinized blood samples were collected from healthy volunteers (n=3, aged 30–35). Whole blood samples were diluted 1:1 with PBS, layered onto Ficoll-Paque (1.077 g/ml) at a volume ratio of 2:1 and centrifuged at 400 g for 30 min at 20°C. The PBMCs layer was collected and washed twice at 1500 rpm for 10 min with RPMI-1640 medium. The supernatant was removed and the cells were resuspended in RPMI-1640 medium containing 10% FBS, 1% penicillin and 100 μg/ml streptomycin. Cell viability was tested (>98% viable cells) using the trypan-blue dye exclusion method.

### Umbelliprenin preparation

Umbelliprenin (C_24_H_30_O_3_, MW: 366) was synthesized as previously described [17]. Umbelliprenin was first dissolved in DMSO, and then this stock was dilluted in CM10 media at 37ºC immediately before use to obtain a maximum DMSO concentration of 0.5% (v/v).

### MTT assay

Exponentially growing cells (T75 flasks) of A549 (5000 cells/well), and QU-DB (7000 cells/well) as well as PBMNCs (1 x 10^5^ cells/well) were cultured onto 96-well plates in RPMI-1640 containing 10% FBS. Cells were allowed to attach for 24 h before treatment. Then, lung cancer cell lines were treated with fresh medium containing umbelliprenin at different concentrations (10, 20, 50, 100, 200 μM). For PBMNCs, media containing umbelliprenin were added at the time of cell seeding in culture plates. ConA (2.5 μg/ml) treated PBMNCs were used as a positive control for umbelliprenin. 0.5% DMSO and untreated cells were used as the control of solvent and negative control, respectively, in all experiments. Wells containing medium with different concentrations of umbelliprenin without cells were used as the control of compound. The plates were incubated for 24, 48 and 72 h. Then, 5 mg/ml MTT (sigma) was added into each well, and the plates were incubated for 4 h (37°C, 5% CO_2_). Media containing MTT were removed and DMSO was added in each well to dissolve the formazan crystals. Absorbance of the wells was read at 570 nm (A_570_) after 30 min of incubation at room temperature (RT). At least three independent experiments were done in triplicate for each experiment.

### Annexin V/PI assay on lung cancer cell lines

To determine the extent of apoptosis and necrosis as well as total cell death (apoptosis + necrosis) induced by umbelliprenin on human lung cancer cell lines, annexin V/PI staining (FITC Annexin V Apoptosis Detection Kit I, BD Pharmingen, USA) was used. Briefly, the cells in the exponential phase were treated with IC_50_ concentration of umbelliprenin for each cell lines. After 48 h, the cells were trypsinized [18-20] (0.25% trypsin with a longer incubation time was used for gentle dissociation of the attached cells), and washed twice with PBS. The pellets were resuspended in 1X binding buffer. After the addition of annexin V-FITC, the cells were incubated for 15 min at RT in the dark. PI dye was added at the last 5 min of total incubation time. Flowcytometry was done within one hour using FACSCalibur flow cytometer (BD Biosciences, USA).

### Statistical analysis

Dose–response curves and IC_50_ values were determined using Curve Expert software, ver.1.3 and expressed as mean *±* standard deviation (SD) from at least three independent experiments. Statistical tests including One-way ANOVA, Tukey multiple comparison or unpaired Student’s *t* tests were performed using GraphPad Prism, ver.5 software. A *p* value of less than 0.05 was considered as significant.

## Results

### MTT assay on lung cancer cell lines

**Figure 1:**
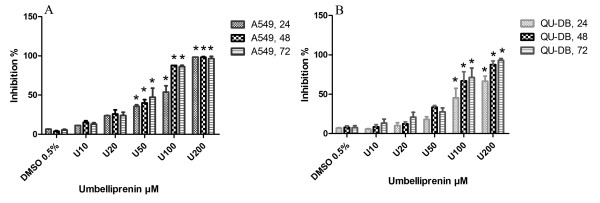
**(A) A549 cells and (B) QU-DB cells after 24, 48, and 72 h incubation with umbelliprenin.** One way ANOVA and Tukey's Multiple Comparison Tests revealed a significant difference between umbelliprenin treated and 0.5% DMSO treated cells. (*) Represents statistical significance (*p<* 0.05).

Using MTT assay, we demonstrated that umbelliprenin has antitumor activity on both A549 and QU-DB cell lines, and that activity was dose and time-dependent. The umbelliprenin concentration resulting in 50% of cell cytotoxicity/proliferation inhibition was considered as IC_50_. These values for A549 and QU-DB were found to be 52 ± 1.97 and 47 ± 5.3 μM, respectively. The data represent the mean ± SD of at least three independent experiments done in triplicate. One way ANOVA analysis revealed a significant difference between umbelliprenin treated cells and 0.5% DMSO treated cells in all experiments (*p<* 0.05). Figure 1 displays the summary of these experiments on both A549 and QU-DB cells.

### MTT assay on PBMNCs

**Figure 2:**
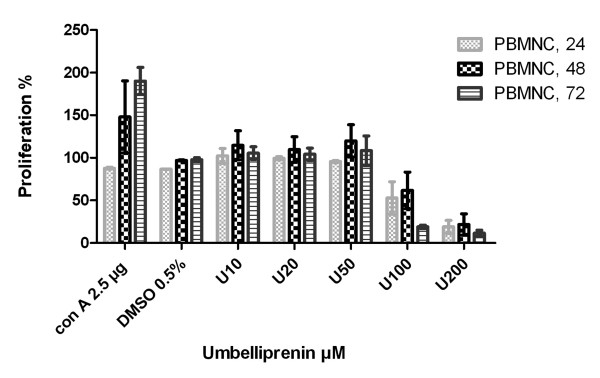
**PBMNCs after 24, 48, and 72 h incubation with different concentrations of umbelliprenin.** At 10, 20, and 50 μM, umbelliprenin showed no cytotoxicity or a mild proliferation effects on PBMNCs, but at higher doses it showed cytotoxic/anti-proliferative effects. 0.5% DMSO was used as the solvent control and Con A 2.5 μg/ml as a positive control for proliferation.

PBMNCs were used to determine the effects of umbelliprenin on a normal, immune cell. In contrast to suppressory effects of umbelliprenin at 10, 20, and 50 μM on lung cancer cell lines, these concentrations caused no inhibition on PBMNCs with even a higher proliferative index compared to DMSO treated cell. However, at higher doses (100 and 200 μM), umbelliprenin showed some inhibitory activity on PBMNCs. Figure 2 indicates the summary of these experiments on PBMNCs.

### Annexin V/PI cell death (apoptosis + necrosis) and trypan blue staining on lung cancer cell lines

**Figure 3:**
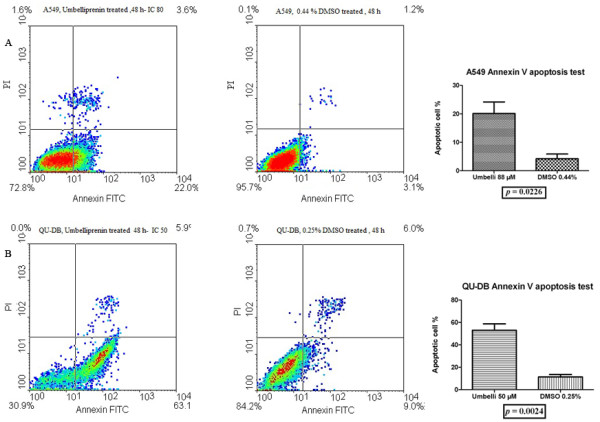
**Annexin V/PI staining. (A) A549 cell treated by umbelliprenin or DMSO after 48 h.** The apoptosis rate was significantly different between umbelliprenin treated A549 cells compared to DMSO treated cells at IC_80_ of umbelliprenin (*p*=0.022). (**B**) QU-DB cell was treated by umbelliprenin or DMSO after 48 h. Umbelliprenin induced apoptosis in QU-DB cells at IC_50_ (50 μM) value after 48 h (*p*=0.002). Numbers adjacent to outlined squares indicate the percentage of cells in each quadrant. LL: Viable cells (Annexin V −/ PI −), LR: early apoptotic cells (Annexin V +/ PI −), UL: late necrotic cells (Annexin V −/ PI +) and UR: late apoptotic/necrotic cells (Annexin V +/ PI +).

For flow cytometry assays, cells were initially studied at their IC_50_ values estimated using MTT. Annexin V/PI staining at IC_50_ of umbelliprenin as compared to DMSO treated controls after 48 h did not show any statistically significant apoptosis or necrosis for A549 cell line. A statistically significant cell death compared to controls was observed after increasing umbelliprenin concentration to IC_80_ (88 μM). The predominant cell death was apoptosis. QU-DB cells were more susceptible to death induced by umbelliprenin than A549 cells, as they showed 50% cell death after 48 h treatment at IC_50_ (50 μM). The dominant cell death was also apoptosis. Figure 3 shows the result of annexin V/PI experiments on both A549 and QU-DB cell lines. Annexin V positive cells detected on FL1, indicated the apoptotic cells. PI positive cells were detected on FL2 and showed death by the necrosis. As it is indicated in Figure 3, the total death rate, either apoptosis or necrosis, at IC_50_ after 48 h for QU-DB was 46.98% ± 8.7% (An^+^ = 41.7%, An^+^/PI^+^ = 4.5%, and PI^+^ = 0.7%), and for A549 at IC_80_ after 48 h was 17.22% ± 6.29 (An^+^ = 15.9%, An^+^/PI^+^ = 0.39%, and PI^+^ = 1.74%). These values are the subtraction of the death rate in the control group from treated group in each quadrant. The percentages of PI positive cells were less than 5% for both cell lines, and the dominant cell death type was apoptosis.

Our flowcytometry results were in accordance with trypan blue viability test. At IC_50_ value, approximately half of QU-DB cells were dead, however, even at IC_80_, more than 50% of A549 cells were still viable in comparison to controls.

## Discussion

Lung cancers are divided into small cell lung carcinoma and non-small cell lung cancer (NSCLC). The majority of lung cancer cases (about 80%) are NSCLC [1]. There are three major subtypes for NSCLC: adenocarcinoma, squamous cell and large call carcinoma (LCC). The latter has the poorest prognosis of NSCLC, followed by adenocarcinoma [21].

Umbelliprenin has been recently recognized as a compound with antitumor activities [9,10]. The data regarding its effects on lung cancer are limited to a few studies [9]. Here we evaluated the effects of this compound on lung cancer cell lines QU-DB and A549 as well as PBMNCs from healthy individuals using MTT assay. Treatment of A549 and QU-DB cells with umbelliprenin at different doses and intervals showed that umbelliprenin significantly suppressed lung cancer cell lines, and this suppression was in a time-dose dependent manner. These lung cancer cell lines showed approximately similar IC_50_ values (≈50μM) using MTT assay. However, we observed the proliferative effects of umbelliprenin on PBMNCs at 10, 20, and 50 μM doses.

To realize whether the decreased MTT reduction of cancer cell lines caused by umbelliprenin is due to cell death or proliferation inhibition, we analyzed cell death using annexin V/PI staining that simultaneously quantifies apoptosis (Annexin V+) and necrosis (PI+). Apoptosis was added up to necrosis to quantify the total death rate. In this study, the percentage of necrosis was less than 5% in all experiments. Consistent with our MTT assay, QU-DB cells showed approximately 50% cell death at IC_50_ concentration after 48 h of umbelliprenin treatment compared to DMSO control cells (*p*=0.002). The total death rate induced by umbelliprenin was 46.98% ± 8.7%, of which 41.7% (An^+^), 4.5% (An^+^/PI^+^), and 0.7% (PI^+^) were related to early apoptosis, late apoptosis and necrosis, respectively. The pattern of cell death was in favor of apoptosis than necrosis. For A549 cell line, we did not observe a significant difference in death rate between treated and untreated cells until we increased the concentration of umbelliprenin to IC_80_ (*p*=0.022). The dominant cell death pattern in this experiment was also apoptosis. The total death rate induced by umbelliprenin was 17.22 ± 6.29, of which early apoptosis, late apoptosis and necrosis patterns constituted 15.9%, 0.39%, and 1.74% death cell types, respectively. These flowcytometry results were partly in agreement with trypan blue viability test in which the number of dead cells was higher in QU-DB than A549 at IC_50_ value calculated using MTT colorimetric assay. Our results indicate that umbelliprenin probably suppresses these cell lines with different pathways. Umbelliprenin is more cytotoxic against QU-DB, a large cell lung cancer cell line compared to A549 adenocarcinoma cell line.

Anti-cancer compounds have been divided into two broad categories according to their ability to damage DNA (cytotoxic), and/or inhibiting either nucleotide biosynthesis or mitosis (anti-proliferative) [22]. Umbelliprenin is structurally related to coumarin family [8]. Coumarin family has been shown both anti-proliferative and cytotoxic effects in different cell lines and situations. Coumarins can exert anti-proliferative effects through inhibition of tubulin polymerization, and induction of cell cycle arrest at G2/M transition of mitotic cell cycle [23]. Coumarins can also induce apoptosis and cell death by mitochondrial dependent and independent mechanisms. They may increase the reactive oxygen species level and release of cytochrome *c* to the cytosol. Several transcription factors, pro-apoptotic and apoptotic molecules are involved in these pathways. For example, it has been shown that apoptotic effects of a coumarin on A549 cells is mediated through pro-apoptotic function of NF-kB by induction of Bax and p53 expression, and reduction of several signaling molecules such as Erk1/Erk2, p38 and JNK [24].

Data regarding the molecular mechanisms of umbelliprenin, itself, are limited. The presence of the aliphatic sesquiterpenoid group linked at C7-OH is related to umbelliprenin cytotoxic effects, but the main mechanism in this regard is unknown [9]. Along with umbelliprenins’ cytotoxicity effects on various cancer cell lines, its anti-proliferative effects on M4Beu cells via cell cycle arrest in G1 phase has been shown before [9]. Other cancer chemopreventive activity of umbelliprenin can be related to its anti-oxidant activity [14] and its anti-inflammatory effects. Umbelliprenin can inhibit the function of 5-lipoxygenase and reduce the production of lipoxygenase carcinogenic products [25]. All of known functions of this compound can lead us to further understanding of its anti-cancer effects.

The apoptotic and anti-proliferative effects of umbelliprenin on two lung cancer cell lines were demonstrated in the present study using MTT and annexin V/PI assays; some limitations still exist. Pairing of MTT and annexin V/PI methods with other tests such as cell cycle analysis by DNA content using PI staining and RNase A [9], or laser scanning confocal microscopy which monitors several mitochondrial events during apoptosis such as permeability transition and Ca2+ fluxes might be further informative regarding anti-proliferative or cytotoxic effects of umbelliprenin [26]. It will be also interesting to uncover the signalling pathways which promote umbelliprenin-induced apoptosis in lung cancer in future studies.

## Conclusions

Using MTT assay and annexin V/PI staining, we demonstrated that umbelliprenin can induce apoptosis in both QU-DB large cell lung cancer and A549 adenocarcinoma cell line but at different doses. The ability of umbelliprenin to induce lymphocyte proliferation along with the induction of apoptosis or inhibition of proliferation in cancer cells makes this compound an attractive compound with fewer side effects for future anti-cancer studies.

## Abbreviations

MTT: Methyl thiazolely diphenyl-tetrazolium bromide; PBMNCs: Peripheral blood mononuclear cells; IC: Index of cytotoxicity; OD: Optical density; PI: Propidium iodide; FBS: Fetal bovine serum; An: Annexin V.

## Competing interests

The authors declare that they have no competing interests.

## Authors’ contributions

The role of each author: NK (nkhaghan@sums.ac.ir): Design, data acquisition and analysis, and drafting the manuscript ZM and AG (ghaderia@gmail.com): Design, data analysis, revising the manuscript, and final approval of the manuscript. MR (RamezaniM@mums.ac.ir): Design, revising the manuscript, final approval of the manuscript. NE (erfanin@sums.ac.ir): Data acquisition. All authors read and approved the final manuscript.
